# Stress Radiograph Confirmation of Translational Instability After Cruciate-Retaining Total Knee Arthroplasty

**DOI:** 10.5435/JAAOSGlobal-D-22-00062

**Published:** 2022-04-15

**Authors:** Darshan Shah, Jordan Hauschild, Donald Hope, David Vizurraga

**Affiliations:** From the Department of Orthopaedics and Rehabilitation, Brooke Army Medical Center, San Antonio, TX.

## Abstract

**Introduction:**

Late rupture of the posterior cruciate ligament (PCL) in cruciate-retaining total knee arthroplasty (TKA) can lead to increased AP instability. This results in increased stress on the medial hamstrings resulting in hamstring-based pain. We looked to identify patients with late PCL failure using a lateral stress radiograph.

**Methods:**

A prospective cohort analysis was completed at a single institution. Eligible patients were divided into two groups based on the amount of anterior knee pain. Pain was defined as a visual analog scale of greater than or equal to 3. Each group completed a visual analog scale, Knee Injury and Osteoarthritis Outcome Score Junior, Patient-Reported Outcome Measurement Information System score and underwent a lateral posterior stress/nonstress radiograph. Amount of posterior translation and posterior tibial slope was measured.

**Results:**

Patients who had painful TKAs at the follow-up had lower Knee Injury and Osteoarthritis Outcome Score Junior (45.86 ± 13.52 versus 78.00 ± 13.26 *P* < 0.001). Those patients were also found to have significantly higher posterior tibial translation with stress radiograph (6.89 ± 1.874 versus 3.91 ± 2.15 mm *P* < 0.001) and significantly increased tibial slope (6.51 ± 2.37° versus 3.98 ± 1.79°, *P* = 0.004). Seven of the 14 patients in the pain group underwent revision surgery, with 6 patients found to have incompetent PCLs.

**Discussion:**

Patients with increased AP translation and increased posterior tibial slope after cruciate-retaining TKA are likely to have worse pain and outcome measures.

The benchmark in the treatment of knee osteoarthritis remains total knee arthroplasty (TKA). Each year more than 750,000 TKAs are done in the United States alone.^1^ Unfortunately, patient satisfaction has varied between 82% in younger patients and 91% in older patients.^2,3^ There are two main design types for TKA, the cruciate-retaining (CR) and posterior stabilized TKAs. One of the leading causes of revision for all types of total knees remains instability, with the overall rate of revision for all causes at 15 years being between approximately 10% and 23%.^4,5,6,7^

The posterior cruciate ligament (PCL) theoretically helps maintain normal knee kinematics by decreasing posterior translation, which allows the knee to maintain normal femoral rollback, decreases the stress over the femorotibial interface in CR designs, and plays a role in balancing the flexion gap.^8-10^ Multiple studies have shown either no difference or better survivorship in patients who received CR TKAs compared with posterior stabilized TKA.^4,6,11^

A risk with the CR design is the eventual rupture or attenuation of the PCL. In a study of 530 patients with PCL retaining TKAs, 13 were found to have PCL ruptures during intraoperative examination at the time of revision surgery. This demonstrates a rate of approximately 2.45% for PCL failure leading to instability that is notable enough to warrant revision knee arthroplasty.^12,13^

Patients reporting flexion instability often feel as though their knee never felt well after surgery and often experience recurrent knee effusions, pain over the pes anserine tendons, and tenderness over the retinacular tissues.^14^ Physical examination findings include a posterior sag when the patient is supine and the knee is held at 90° and a tibial distraction stress test.^14,15^ These finds are likely due to the traumatic fatigue failure of the sagittal plane stabilizers of the knee.^14,16^ The cause of this pain is still not well understood, and diagnostic tools are limited.^15,17,18^ Currently, limited literature exists on stress radiographs as a tool to help correlate the findings associated with flexion instability and potential PCL insufficiency.^19,20^

The goal of this study was to determine whether we can correlate anteromedial knee pain/knee instability with radiographic evidence of posterior tibial instability in patients who underwent CR TKA. Our hypothesis is that patients with anteromedial knee pain will have increased posterior translation on stress radiographs.

## Methods

This study was a single-center prospective study in which patients who were greater than 6 months from their CR TKA were evaluated for posterior instability. The study was approved by our institution’s Institutional Review Board. Written consent was obtained from all participants.

### Patients and Procedures

Between June 2019 and January 2020, 26 individuals (27 knees) with CR TKAs were enrolled in this study. Inclusion criteria included being greater than 6 months out from primary CR TKA. All procedures included were completed by fellowship-trained adult reconstruction surgeons. Exclusion criteria included age less than 45 years or greater than 90 years, evidence of perihardware lucency, obvious failure of tibial or femoral hardware, or periprosthetic fracture. Pain was defined as a visual analog scale value of 3 or greater.

All patients who were enrolled underwent 90° bent knee stress and nonstress radiographs to assess for posterior translational instability. The radiograph was done as described by Jung et al.^20^ Participants were seated in a chair with their knees bent to 90°. Their heel was resting against a bar. For the nonstress radiograph, a lateral with no muscle activation was obtained. For the stress radiograph, the patients were instructed to slide their heel backward into the metal bar to induce hamstring contraction. Although the hamstring was contracting, a lateral radiograph of the knee was obtained.

Tibial translation was determined based on the distance from the posterior condyles to the posterior aspect of the tibial baseplate. The tibial slope was measured on the nonstress radiograph of the knee. All radiographic review was completed by a single interpreter (J.H.) who was blinded to both pain score and patient-related outcome measures.

### Outcome Measures

At the time of enrollment, patient outcomes were measured using Knee Injury and Osteoarthritis Outcome-JR (KOOS-JR), Patient-Reported Outcomes Measurement Information System (PROMIS), and a visual analog scale (VAS).^21,22^

### Statistical Analysis

According to sample size calculations, four knees were needed in each study arm to detect a statistically significant difference in posterior translation.^20,23^

All data were analyzed using SPSS version 22 (IBM SPSS Statistics for Windows, version 22.0; IBM). Descriptive statistics are presented for both groups (pain and no pain) in the form of the number of occurrences and percentages or as the mean and SD. For bivariate analyses of continuous variables, a Wilcoxon/Kruskal-Wallis test for nonparametric data with a one-way chi-square approximation was used. Significance was set at *P* < 0.05. The Pearson chi-square test less than 0.05 was considered to represent a notable difference in categorical variables. The Fischer exact test of less than 0.05 was used when any group had a value less than 5.

## Results

The average age of the patients in the pain group was 64.07 ± 8.67 years (n = 14) compared with 63.15 ± 5.70 years (n = 13) (Table 1). No significant difference was observed in age (*P* = 0.75), BMI (*P* = 0.47), laterality (*P* = 0.49), insert type (*P* = 0.33), or sex (*P* = 0.12) (Table 1). A significant difference was observed (*P* = 0.007) in time since the index procedure, with the no pain group having significantly shorter time from surgery with a mean of 40.5 months versus 79.6 months for the no pain group (Table 1).

**Table 1:** Patient Characteristics

Characteristic	Pain	No Pain	*P* Value^a^
Age	64.1 ± 7.8.7	63.2 ± 5.7	0.75
BMI	30.9 ± 5.2	29.6 ± 4.4	0.47
Laterality (L:R)	9:5	7:6	0.49
Insert type (CS:CR)	6:8	8:5	0.33
Months since surgery	79.6 ± 39.8	40.5 ± 29.0	0.007
Sex (F:M)	6:8	2:11	0.12

BMI = body mass index, CS = condylar stabilized

Anterior constrained (AC) versus standard cruciate retaining (CR) polyethylene insert.

a Values were considered significantly different if *P* < 0.05.

Patient-related outcome measures showed a significant difference in the mean KOOS-JR score for the pain group versus the no pain group (45.86 ± 13.52 versus 78.00 ± 13.26 (*P* < 0.001), respectively) (Table 2). No notable differences were observed in PROMIS physical or mental health scores.

**Table 2:** Results of Bivariate Analysis of Pain Versus No Pain Cruciate Retaining Total Knee Arthroplasty

Characteristic	Pain	No Pain	*P* Value
Current VAS	6.2 ± 1.5	0.8 ± 0.9	<0.001^a^
VAS max	8.7 ± 1.5	2.9 ± 2.4	<0.001^a^
PROMIS T-score physical^b^	45.3 ± 9.6	51.0 ± 6.1	0.077
PROMIS T-score mental^b^	58.4 ± 12.9	55.2 ± 8.7	0.454
KOOS-JR^c^	45.9 ± 13.5	78.0 ± 13.2	<0.001^a^
Posterior tibial translation (mm)	6.89 ± 1.87	3.91 ± 2.15	<0.001^a^
Posterior tibial slope (°)	6.89 ± 1.87	3.91 ± 2.15	0.004^a^

KOOS-JR = Knee Injury and Osteoarthritis Outcome Score-Junior, PROMIS = Patient-Reported Outcome Measurement Information System, VAS = visual analog scale

Values are given as mean ± SD.

a Significance was set at *P* < 0.05.

b The values shown are for the converted T-scores of the PROMIS measure.

c The value shown is the KOOS-JR converted score.

The mean tibial translation for the pain group was 6.89 ± 1.87 mm versus 3.91 ± 2.15 mm for the control group (*P* < 0.001). The average tibial slope for the pain group was 6.51 ± 2.37°. The mean tibial slope for the control group was 3.98 ± 1.79° (*P* = 0.004) (Table 2). In the pain group, there were seven revision surgeries for continued pain/instability and six PCLs were found to be incompetent at the time of surgery. Of those patients who had revision surgery, they all had at least a three-point improvement in the VAS score postoperatively at their most recent follow-up.

## Discussion

**Figure 1:**
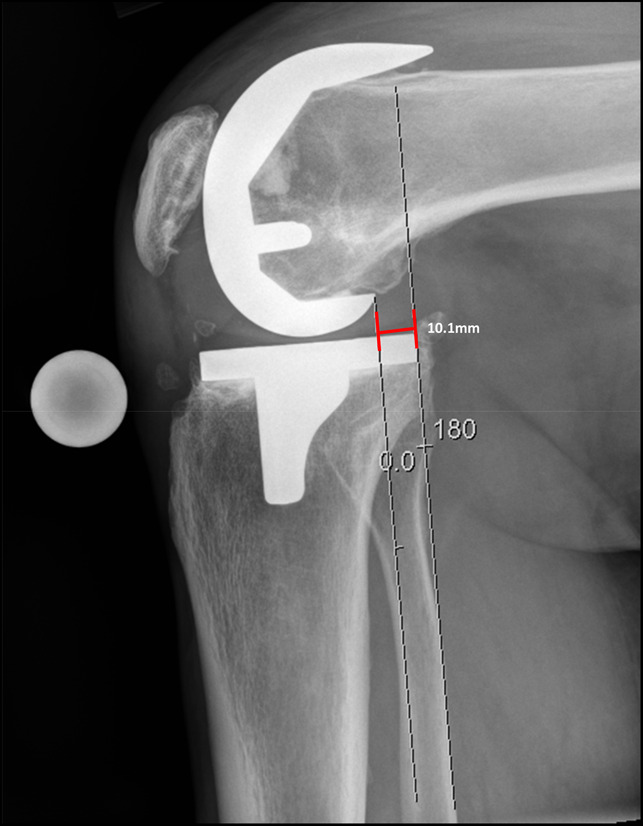
Prestress lateral radiograph of the knee demonstrating 10.1 mm of space between the posterior femoral condyle and the posterior aspect of the tibial baseplate.

**Figure 2:**
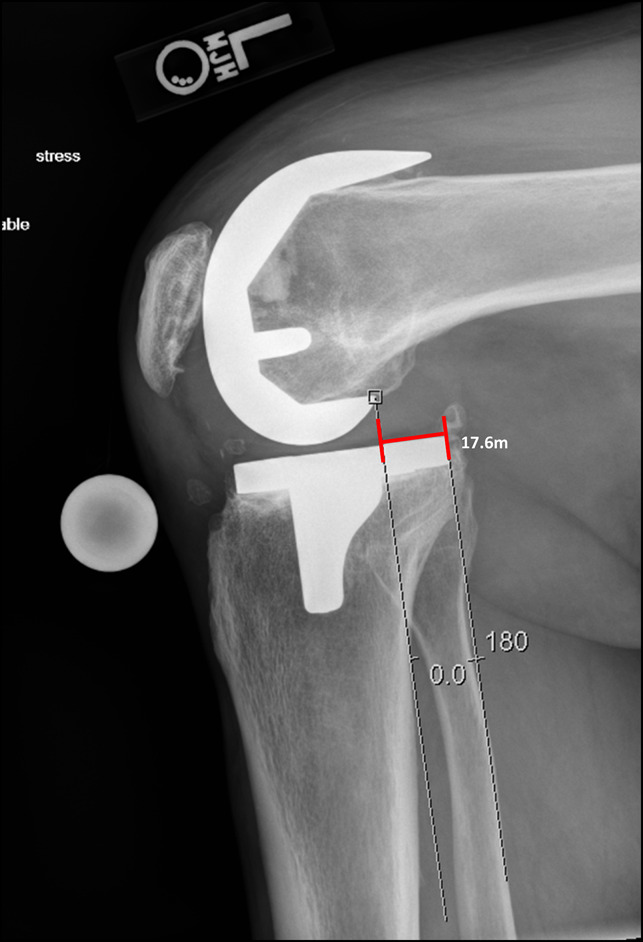
Poststress lateral of the knee demonstrating 17.6 mm of gap between the posterior femoral condyle and the posterior aspect of the tibial baseplate. Stress was induced by the patient actively firing their hamstrings while image was obtained.

This was a single-center prospective study in which patients who were greater than 6 months postoperatively from a CR TKA were evaluated for instability. We found that patients who were experiencing pain and the clinical symptoms suggestive of PCL instability had significantly increased posterior tibial translation on stress radiographic evaluation (Figures 1 and 2). Those with pain had posterior translation 6.59 ± 1.78 mm versus 3.66 ± 1.78 mm in patients reporting no pain or clinical symptoms of instability (*P* < 0.05) (Table 2). Furthermore, patients with pain had a significantly increased posterior slope compared with patients without pain (6.51 ± 2.37° versus 3.98 ± 2.15°, *P* < 0.001) (Table 2). Those individuals in our study having significant pain and clinical findings of AP instability also had significantly lower outcome scores on the KOOS-JR and were significantly further out from surgery than the control group (Tables 1 and 2). No notable difference was observed in the physical or mental health portion of PROMIS.

Knee instability is the second leading cause of TKA revisions in the first 5 years and accounts for 8% to 22% of all revision procedures. Studies have shown that the rate of late PCL rupture in CR knees can be as high as 2.45%.^12,24,25^ Even in patients with an intact PCL, clinical instability can still be present if their PCL is clinically incompetent. These patients report recurrent knee effusions, pain over the pes anserine tendons, and tenderness over the retinacular tissues. In addition, many of these patients also report that their knee never felt well after their index procedures or will have a traumatic event where their knee begins to feel unstable.^14,16^ The lack of improvement after the index procedure for some individuals remains a mystery. Several grading systems exist for AP translation of the knee; however, none of these are agreed on.

Previous studies had looked at clinical examinations, such as anterior drawer techniques, where instability is graded on a mild, moderate, and marked scale, where translations are 5 mm, 6 to 10 mm, and >1 cm, respectively.^4^ A recent study by Murer et al^19^ used stress radiographs to assess posterior instability in patients who had a painful total knee arthroplasty. Their findings were in agreement with the findings of this study that those patients with increased AP instability had incompetent PCLs and increased translation on stress radiography. Our study suggests that individuals with clinically incompetent PCLs can be identified based on their clinical examination and demonstrated that this correlates with the amount of tibial translation on stress radiographs. This has the possibility to offer clinicians a more reliable and reproducible way of demonstrating PCL insufficiency in the correct patient populations.

Several previous authors have demonstrated the importance of posterior tibial slope on the PCL insertion and have demonstrated that the increased posterior tibial slope increases the amount of the PCL insertion that is resected.^24,26,27^ Sacrificing the PCL insertion markedly decreases the tensile strength of the PCL and could lead to rupture and/or PCL insufficiency with time.^28^ Unfortunately, lower posterior tibial slopes lead to increased strain on the retained PCL, and therefore, most CR knees preferentially create the increased posterior slope to help maintain normal knee kinematics and decrease the strain on the retained PCL.^29^ The patients in our study had the increased posterior slope suggesting that this may be a contributing factor to PCL insufficiency in CR knees.

Weakness in the PCL with increased insertion recession could explain the temporal relationship seen in this study. Although the flexion gap may have been balanced at the time of surgery and for some time following, the PCL eventually failed. Once the PCL failed the flexion gap no longer remained balanced allowing increased sagittal plane translation.^30,31^

Additional longitudinal studies would be needed to characterize this relationship and identify additional variables which could explain these findings. Because implant technology has advanced, the idea that all stability in TKAs comes from soft tissue has been questioned. Newer implants such as the medial pivot and anterior lipped implants have been shown to provide sagittal plane stability even when the PCL is resected through increased congruency.^32,33,34,35^ Regardless of the implant used, the flexion gap must be balanced to allow the knee to function properly and have adequate sagittal plane stability.^36-38^ The patients in this study initially had balanced flexion and extension gaps and that coupled with the congruence was able to provide the sagittal plane stability needed to avoid the flexion instability that was seen (Table 2). Then whether through trauma or attrition, the PCL failed leading to an unbalanced flexion gap leading to the observed sagittal plane instability.^9,10^

This study has several limitations. First, it was single-center study, and the number of participants was small with only 26 participants in total. However, even with this small sample size, we were able to reach notable values which suggest an important relationship between clinical evidence of PCL insufficiency and radiographic evidence of PCL incompetence. Second, there was not a standardized amount of posterior force used while performing our stress radiographs. Although this may be a limitation, we do not feel that standardization of this parameter is necessarily beneficial because the strength and competence of the patient's musculature and ligamentous structures play a crucial role in the feelings of instability. Finally, there were several different implant types spanning a variety of manufacturers. Additional studies would be needed to increase the sample size and standardize implant types or to perform a subgroup analysis of the different implant types and polyethylene inserts to determine whether these play a role in the development and presentation of PCL instability. Additional studies would be needed to determine whether patients with clinical and radiographic findings of instability have PCL ruptures or incompetence at the time of revision surgery and how their symptoms and functional outcome scores change after revision.
